# Deep Transfer Learning Method Using Self-Pixel and Global Channel Attentive Regularization

**DOI:** 10.3390/s24113522

**Published:** 2024-05-30

**Authors:** Changhee Kang, Sang-ug Kang

**Affiliations:** Department of Computer Science, Sangmyung University, Seoul 03016, Republic of Korea; 202032028@sangmyung.kr

**Keywords:** deep transfer learning, knowledge distillation, regularization

## Abstract

The purpose of this paper is to propose a novel transfer learning regularization method based on knowledge distillation. Recently, transfer learning methods have been used in various fields. However, problems such as knowledge loss still occur during the process of transfer learning to a new target dataset. To solve these problems, there are various regularization methods based on knowledge distillation techniques. In this paper, we propose a transfer learning regularization method based on feature map alignment used in the field of knowledge distillation. The proposed method is composed of two attention-based submodules: self-pixel attention (SPA) and global channel attention (GCA). The self-pixel attention submodule utilizes both the feature maps of the source and target models, so that it provides an opportunity to jointly consider the features of the target and the knowledge of the source. The global channel attention submodule determines the importance of channels through all layers, unlike the existing methods that calculate these only within a single layer. Accordingly, transfer learning regularization is performed by considering both the interior of each single layer and the depth of the entire layer. Consequently, the proposed method using both of these submodules showed overall improved classification accuracy than the existing methods in classification experiments on commonly used datasets.

## 1. Introduction

In recent computer vision literature, deep learning approaches are being used in various fields. Hussein et al. [1] applied a deep network to the medical field to classify lung and pancreatic tumors. Ramesh et al. [2] devised a text-to-image generator that interprets the meaning of input text and then creates an image containing the interpreted meaning. Also, Feng et al. [3] proposed an object segmentation method that divides objects such as pedestrians, vehicles, roads, and traffic lights for autonomous driving. Kang et al. [4] replaced traditional denoising image filters with a single recursive neural network to remove various types of unwanted signals. For most of these tasks, supervised learning performed better when the number of training datasets was sufficient, as demonstrated by Orabona et al. in [5] with the well-known MNIST dataset. However, since data collection and labeling are time consuming and costly, transfer learning approaches have emerged.

Transfer learning aims to perform new target tasks on small-scale data by leveraging deep neural network knowledge pretrained on large-scale data, as shown in [6,7]. For example, various deep models like ResNet [8], VGG [9] were pretrained on large-scale datasets such as ImageNet [10], and then utilized for new target tasks. Using a pretrained model, also called the source model, as a starting point, you can use fine-tuning techniques to transform it into a newly trained model for a new target task. Some of the weights of the source model are changed during separate training sessions using a new target dataset in order to create new target models for new tasks [11,12,13,14]. In general, the performance of fine-tuning techniques, such as convergence speed and prediction accuracy, is better than traditional supervised learning. For example, Ng et al. [15] showed about 16% higher emotion recognition accuracy, Zhao [16] achieved 7.2% better classification results, and Mohammadian et al. [17] attained about 12.1% improvement over the conventional approach [18] in diabetes diagnosis. However, there are two problems with the fine-tuning approaches. First, the distribution of source and target datasets should be similar. Wang et al. [19] defined negative transfer learning as the phenomenon in which source knowledge interferes with target learning when the source and target domains are not sufficiently similar. It was demonstrated by showing that the larger the gap between the source and target domains, the lower the transfer learning performance. Second, fine-tuning the target model often loses some useful knowledge for the target task learned from the source model, even when there is only a slight distributional difference between the source and target datasets, as demonstrated in [20].

To cope with these problems, $L^{2}$ regularization is applied to the weights of target model during the fine-tuning process. Li et al. [21] proposed the $L^{2}-SP$ method, which encourages the weights of the fine-tuned target model to be similar to those of the source model, called the starting point (SP). This $L^{2}-SP$ regularization method showed about 0.8% to 8.6% better results than the vanilla $L^{2}$ regularization method [21,22]. However, weight regularization approaches often fail to converge the target model or often lose useful knowledge learned from the source model, as it is difficult to find the appropriate regularization strength due to optimization sensitivity [20]. To address this problem, Hinton et al. [23] proposed the knowledge distillation method, which extracts only the necessary knowledge from the source model, rather than all of it, and transfers it to the target model. It also allows for different source and target model structures, typically large for the source and simple for the target. Therefore, the target model is trained utilizing feature maps of the source model, instead of weights, as in [24]. Therefore, both source model and target model are necessary during the training session of the target model. Utilizing the entire spatial area and all channels of the feature map is sometimes not effective, so methods have been proposed to use only the parts that are actually influential [25,26]. Mirzadeh et al. [25] proposed a distillation method using a teacher assistant model, which is an intermediate size between the teacher and student models. Li et al. [26] added a 1×1 convolution layer to each specific layer of the student model to make its feature map similar to the corresponding feature map of the teacher model. Li and Hoiem [27] proposed a transfer learning method called LWF (learning without forgetting), which can learn a new task while retaining the knowledge and capabilities originally learned. The LWF has integrated the knowledge distillation method into the transfer learning process so that it is possible to learn without forgetting the original knowledge, even when using only dataset for a new task. Li et al. [28] proposed the DELTA (deep learning transfer using feature map with attention) method, which assigns attention scores to feature maps based on the LWF method.

The DELTA method [28] determines the importance of each filter by calculating the loss value using a feature extractor model($L^{2}-FE$) that determines the useful of the filter. $L^{2}-FE$ is trained only the fully-connected layers of the source model using the target data. After filling each filter of a specific layer from the $L^{2}-FE$ model with a value of 0, the importance of the filters is calculated according to the changing loss value between prediction and label. Through the calculated importance of source model filters, the target model trained with a regularization method using an attention mechanism [29,30] that gives weights to filters containing useful knowledge in the source model. Xie et al. [31] proposed attentive feature alignment (AFA) based on a knowledge distillation paradigm [24] similar to DELTA. AFA extracts attention weights in the spatial and channel information related to the target from the feature map extracted from the source model through the additional submodule networks. While DELTA calculates the importance of a convolution filter using the subtraction in loss values, AFA calculates the importance of a convolution filter using a submodule network defined as an attentive module. The attentive module consists of two types of modules that receive the feature map extracted from the convolutional layer and calculate the importance of the convolutional filter by reflecting spatial or channel information. Compared to the DELTA method, AFA considers attention to the spatial information as well as channels and uses a method of calculating weights through a submodule network.

Both the AFA [31] and DELTA [28] methods determine the relative importance of the source model filters in the target models and represent it as real numbers ranging from 0 to 1, all summing to 1. However, the importance comparison is evaluated only within the scope of a single convolution layer, i.e., the same value in different convolution layers has the same importance throughout the target model. Since different convolution layers have different roles, e.g., simple functionalities for input side layers and vice versa, it is natural that each convolution layer has a different impact on the target model. Therefore, the relative importance of a filter should also be determined considering the position of a convolution layer. In this paper, the importance of filters is not compared within a single convolution layer, but across all layers. Thus, we propose a global channel attention module based on the SENet method [32]. In addition, we propose a self-pixel attention module that regularizes with the feature map of the target model as well as the feature map of the source model, which extends the concept of spatial attention module proposed in AFA. Our main contributions are summarized as follows. First, we propose a global channel attention submodule that determines the channel importance of all layers, unlike existing methods that use channel importance only within a single layer. Second, even if only the global channel attention submodule is used, it shows similar or improved performance to existing methods. Third, the proposed regularization method using the two proposed attention submodules shows overall improved classification accuracy and convergence speed compared to existing regularization methods. The contents of this paper are organized as follows. Section 2 explains related works, Section 3 describes the proposed regularization model for transfer learning, Section 4 explains the experimental settings such as the dataset and hyperparameters used in the experiment, and Section 5 describes the experimental results. Finally, Section 6 concludes the paper.

## 2. Related Works

The AFA method [31] uses two submodules: AST (attentive spatial transfer) and ACT (attentive channel transfer), which take the feature map of the source model as input and calculate the attention values for regularized optimization of the target model. The AST module calculates feature-specific attention values and the ACT module outputs channel-specific values. The AST and ACT calculate weighting values for spatial positions and channels of feature maps respectively. Since feature maps are used to regularize the optimization, we need to define the feature map first, as shown in Equation (1).

(1) $$FM_{S\;or\;T}^{i}=f\left(x,W_{S\;or\;T}^{i}\right)$$

where the superscript *i* and the subscripts S,T refer to the *i*’th convolutional layer of the source model or the target model used for regularization, and $W_{S\;or\;T}^{i}$ and *x* denote the weights of *i*’th layer and an input image, respectively. The feature map of the AST network can be derived by flattening the $FM_{S}^{i}$ along the height and width directions using the flatten function $Flat\left(\cdot \right):R^{C\times H\times W}\to R^{C\times (HW)}$, and then average pooling the result along the channels using the function $AvgPool\left(\cdot \right):R^{C\times (HW)}\to R^{1\times (HW)}$. The attention weights $AST^{i}$ can be calculated using Equation (2) and then the AST loss $L_{AST}$ is calculated using Equation (3), respectively.

(2) $$AST^{i}=Softmax\left(FC_{AST}^{i}\left(AvgPool\left(Flat\left(FM_{S}^{i}\right)\right)\right)\right)$$

(3) $$L_{AST}=\sum_{i=1}^{n}\frac{1}{2}{∥AST^{i}\cdot \left(FM_{S}^{i}-FM_{T}^{i}\right)∥}_{F}^{2}$$

where the $FC_{AST}\left(\cdot \right):R^{1\times (HW)}\to R^{HW}$ consists of two fully-connected layers, and *n* denotes the total number of convolutional layers selected to extract feature maps. The difference between $FM_{S}^{i}$ and $FM_{T}^{i}$ is multiplied by the attention weights element by element, and then the Frobenius norm of the vector is computed in order to find the AST loss of the *i*’th convolutional layer. The AST loss $L_{AST}$ is the sum of all the losses of the *n* selected convolutional layers. By minimizing $L_{AST}$, the original knowledge is differentially transferred to the target model. The ACT module weighs the importance of the channel information in the source feature maps. The attention weights $ACT^{i}$ can be calculated using Equation (4).

(4) $$ACT^{i}=Softmax\left(FC_{ACT}^{i}\left(Flat\left(FM_{S}^{i}\right)\right)\right)$$

Unlike the AST submodule, the ACT submodule only applies a flattening function and no average pooling to obtain the transformed feature map [31]. Therefore, the feature map is transformed using the flatten function $Flat\left(\cdot \right):R^{C\times H\times W}\to R^{C\times (HW)}$, and finally, $ACT^{i}$ is determined by $FC_{ACT}\left(\cdot \right):R^{C\times (HW)}\to R^{C}$. The ACT loss $L_{ACT}$ is expressed by Equation (5).

(5) $$L_{ACT}=\sum_{i=1}^{n}\frac{1}{2}ACT^{i}\cdot {∥(FM_{S}^{i}-FM_{T}^{i})∥}_{F}^{2}$$

After calculating the difference between two feature maps, the attention weight values calculated by the ACT module are multiplied by the magnitudes of the channel vectors. The $L_{ACT}$ is obtained by adding all the loss values of the selected *n* convolutional layers. For the whole training period, we devote half of the epochs to AST and the other half to ACT. The transfer learning objective function is as follows.

(6) $$L_{Total}=L_{CE}+\alpha \cdot L_{AST\;or\;ACT}+\beta \cdot WD$$

where $L_{CE}$ is cross-entropy loss function and WD is weight decay. $L^{2}$ regularization is applied to the weights of the fully-connected layers of the target model. The values of α and β are the coefficients used for each loss value.

## 3. The Proposed Method

**Figure 1:**
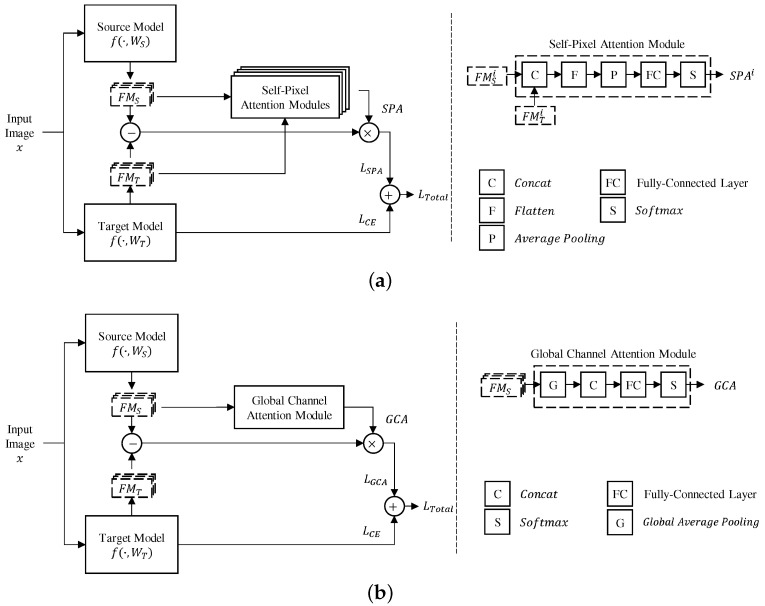
Block diagram of the proposed regularization method: (**a**) Overview of the training process with SPA (Self-Pixel Attention) module. (**b**) Overview of the training process with GCA (Global Channel Attention) module.

The two submodules SPA (self-pixel attention) and GCA (global channel attention) are proposed as shown in Figure 1. The SPA extends the AST in [31] in order to strengthen the related knowledge between the source and the target models by utilizing both their feature maps. The GCA calculates the channel importance of all convolutional layers together, rather than per layer as in [31].

### 3.1. The Self-Pixel Attention Submodule

AFA [31] applied pixel attention to consider the importance of spatial information, and showed improved performance than DELTA [28], which did not consider spatial information. However, the previous pixel attentive module simply utilized only the spatial information of the feature map extracted from the source model to calculate the attention weights. The proposed SPA calculates attention weights by concatenating $FM_{S}^{i}$ and $FM_{T}^{i}$ to exploit the spatial information of the source and target models in the regularization process of training.

(7) $$SPA^{i}=Softmax\left(FC_{SPA}^{i}\left(AvgPool\left(Flat\left(Concat\left(FM_{S}^{i},FM_{T}^{i}\right)\right)\right)\right)\right)$$

The concatenation is performed along the channel direction using the function Concat(·): $R^{C_{S}\times H\times W},R^{C_{T}\times H\times W}\to R^{(C_{S}+C_{T})\times H\times W}$. Flatten and average pooling functions transform the dimension of the feature map to $R^{1\times (HW)}$. The fully-connected layer $FC_{SPA}^{i}\left(\cdot \right)$ followed by the softmax function outputs the attention weights of the *i*’th convolutional layer. Finally, the SPA loss is calculated as in Equation (8), similar to Equation (3).

(8) $$L_{SPA}=\sum_{i=1}^{n}\frac{1}{2}{∥SPA^{i}\left(FM_{S}^{i}-FM_{T}^{i}\right)∥}_{F}^{2}$$

### 3.2. The Global Channel Attention Submodule

Equal attention weight means that the corresponding channels have the same importance in the previous works, regardless of the importance of the convolutional layer that includes them. For example, the deeper layers usually contain more information or knowledge than the shallower layers. Therefore, the proposed GCA calculates the relative importance of channels in all selected convolutional layers.

(9) $$\begin{array}{cc} GCA=\; & Softmax(FC_{GCA}(Concat(\\ \; & GAP\left(FM_{S}^{1}\right),\cdots ,GAP\left(FM_{S}^{n}\right)))) \end{array}$$

The feature map of source model $FM_{S}^{i}$ is the input to the global average pooling function GAP(·): $R^{C\times H\times W}\to R^{C\times 1}$. The final GCA vector is in the space of $R^{C_{1}+C_{2}+\cdots +C_{n}}$. The GCA loss is computed as in Equation (10), similar to Equation (5).

(10) $$L_{GCA}=\sum_{i=1}^{n}\frac{1}{2}GCA^{i}{∥(FM_{S}^{i}-FM_{T}^{i})∥}_{F}^{2}$$

The subtraction between the feature maps of the *i*’th convolutional layer obtained from the source and target models is multiplied for each channel by the GCA obtained through Equation (9). $GCA^{i}$ is a channel attention weight corresponding to the *i*’th convolutional layer and has the size of $R^{C_{i}}$, which is a part of GCA. By applying this GCA loss, the target model is regularized in the channel direction, and in the source model, not only the channel attention of a single layer but also the relative emphasis according to the depth is progressed. The structure of the fully connected layers used in the proposed method can be confirmed by Section 4.2.

### 3.3. Objective Function for Regularization

The optimization process is divided into two stages and is similar to the previous work, AFA [31]. In the first stage, we use a pretrained model such as ImageNet and Places 365, selected according to the nature of the target task, as the source model. The weights of the convolutional layer of the source model are transferred to the target model, and those of the SPA submodule and the fully connected layers of the target model are randomly initialized. The target model is then trained during the first half epochs using the loss function in Equation (11) derived from Equation (6).

(11) $$L_{Total}=L_{CE}\left(f\left(x,W_{T}\right),y\right)+\alpha \cdot L_{SPA}+\beta \cdot WD$$

The $L_{CE}$ is determined by calculating the cross entropy of the predicted and ground distributions. The WD is the $L^{2}$ regularization weight decay applied to the weights of the fully connected layers of the target model. In the second stage, the weights from the target model trained in the first stage are sent back to the source model because they contain better knowledge than the original weights from the source. Then, only the weights of the GCA submodule are initialized randomly, and the loss function in Equation (12) is used to train the target model for the remaining epochs.

(12) $$L_{Total}=L_{CE}\left(f\left(x,W_{T}\right),y\right)+\alpha \cdot L_{GCA}+\beta \cdot WD$$

## 4. Details of the Experiments

### 4.1. Dataset Setup

We evaluate the performance of the proposed method through object and scene classification. For object classification, the source model was pretrained using ImageNet [10], and the target model used Stanford Dogs 120 [33], Caltech 256-30 [34], Caltech 256-60 [34], and CUB-200-2011 [35] datasets. For scene classification, the source model was pretrained utilizing the Places 365 dataset [36], and the target model was trained and tested using the MIT Indoor 67 dataset [37]. The purpose and characteristics of each target dataset are summarized in Table 1. The Stanford Dogs 120 dataset contains puppies by breed, and consists of a total of 20,580 images for 120 breeds. Caltech 256 is an object recognition dataset containing 30,607 real-world images, of different sizes, spanning 257 object classes, consisting of 256 object classes and an additional clutter class. Each class is represented by at least 80 images. Caltech 256-30 randomly selects 50 images from the Caltech 256 dataset and divides them into 30 and 20 training and test images respectively. Similarly, Caltech 256-60 selects 80 images and divides them into 60 and 20. The CUB-200-2011 dataset is a fine-grained classification of birds by breed, with a total of 200 breeds and 11,788 images. MIT Indoor 67 is a dataset of 67 indoor scenes with a total of 15,620 images. However, only 6700 of them are used in this experiment: 5360 for training and 1340 for testing. During the experiment, the images are resized to 256 × 256 and then cropped to 224 × 224 at random locations to use as input to the model. The data configuration and image preprocessing methods were set to be as similar as possible to existing studies.

### 4.2. The Structure of Network and Hyperparameters

Most experiments are performed using the ResNet-101 model [8] pretrained with ImageNet. However, for our experiments on the MIT Indoor 67 dataset, we used the ResNet-50 model because it is the only pretrained model available for the Places 365 dataset. For model optimization, the SGD is used with the momentum set to 0.9 and the batch size set to 64. SPA and GCA are trained sequentially for 4500 iterations each out of a total of 9000 training iterations. The structure of each submodule can be found in Table 2. The initial learning rate is 0.01 and decreases to 1/10 at two points: two-thirds of the way through the SPA training period and two-thirds of the way through the GCA training period, eventually becoming 0.0001. In this experiment, the learning rate decay occurs at 3000 and 7500 iterations. In addition, *r* of the fully-connected layer used in the GCA submodule is set to 4. The weighting factor α, which means the strength of the loss value calculated from the submodules, is set in the range of 0.005 to 0.1 depending on the target dataset. The weighting factor β, the intensity of the $L^{2}$ weight decay, is set to 0.01. The feature maps used as input to the submodules are extracted from a total of four intermediate layers of the source and target models, and are chosen to be the same as in DELTA [28] and AFA [31] for a fair comparison. Additionally, in order to apply the proposed method other than the ResNet models, it is important to select intermediate layers that can well represent the features comprehensively. The selected intermediate layers can be found in Table 3.

## 5. Experimental Results

### 5.1. Performance Comparison

To validate the performance of the proposed method, two experiments are carried out and the results are presented with the mean and standard deviation after five identical experiments. Two experiments are performed using the five types of dataset described in Section 4.1.

**Figure 2:**
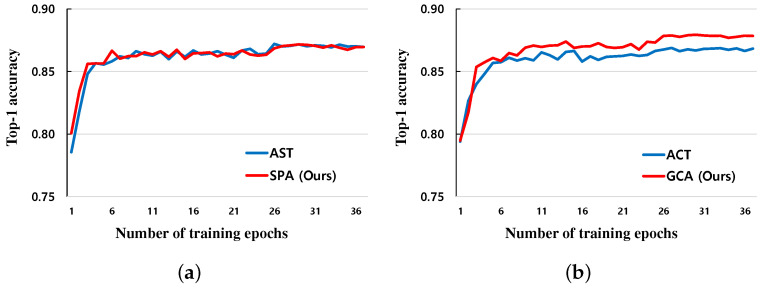
Comparison of top-1 accuracy results with different submodules on Caltech 256-60. (**a**) Comparison between AST [31] and SPA modules. (**b**) Comparison between ACT [31] and GCA modules.

The first experiment compares the proposed method with five existing methods: $L^{2}$, $L^{2}-FE$ [28], $L^{2}-SP$ [21], DELTA [28], and AFA [31]. For all datasets, the proposed regularization method shows an overall improved performance compared to previous transfer learning regularization methods. The classification accuracy for the CUB-200-2011 dataset [35] was boosted by approximately 0.32% in comparison to the AFA method. For the MIT Indoors 67 [37], the improvement is 0.48%, which is quite good for a small number of training data. However, the improvement is relatively small for the Stanford Dogs 120 [33] and Caltech 256-60 [34]. The first experimental results can be confirmed by Table 4. In the second experiment, we compared the performance between SPA and GCA, which are submodules of the proposed method, and AST and ACT, which are submodules of the AFA method. After applying each submodule to the target model one by one, SPA and GCA showed similar or improved classification accuracy results across the board. For most datasets, there was a slight improvement of the SPA over the AST. Regarding the Caltech 256-60, the GCA showed about 0.85% improvement over the ACT. However, for the MIT Indoors 67 dataset, the SPA resulted in 0.18% less accuracy, whereas the GCA yielded an increase by 0.69% over the ACT. The results can be checked through Table 5. In addition, both proposed submodules converged faster than the submodules of the previous AFA method. The SPA module converged quickly but had similar performance, while the GCA module converged faster and had improved performance. The comparison of the convergence speed can be confirmed by Figure 2. According to the results of the first and second experiments, the overall performance was improved when both of two proposed submodules were used. The proposed SPA and GCA submodules showed improved performance in most cases than the existing submodules. Additionally, as shown in Table 5, when only the GCA module was used, the performance was similar or improved compared to the existing methods. Therefore, it is useful and important to utilize the feature maps of the target model and calculate the channel importance across all layers rather than the existing single layer-wise regularization methods.

### 5.2. Ablation Study

We conducted an experiment to assess how the reduction rate of GCA submodules, expressed as *r*, impacts the regularization model. The reduction rate is defined in Section 4.2. Using the ResNet-101 model and the Caltech 256-30 dataset, we measured the object classification accuracy while varying the *r* value of GCA, and the experimental results are shown in Table 6. Experiments have shown that classification accuracy tends to decrease as *r* increases. This is because more information contained in the filter values in the source layers is lost at the bottleneck of the fully connected layer. In this paper, we set the reduction rate to 4, which is the lowest level.

Another experiment was conducted to determine the difference in transfer learning performance based on the training order of the SPA and GCA submodules. The comparison of classification accuracy using the Caltech 256-60 dataset is shown in Table 7. The same as the additional experiment of the AFA [31] method using two submodules for transfer learning, a better result is obtained when the SPA submodule is trained before the GCA submodule. To reflect this result, we trained SPA for the first half of the total training session and then GCA for the remainder of the session.

## 6. Conclusions

In this paper, we propose an improved deep transfer learning regularization method. The proposed method uses a global channel attention submodule that determines the channel importance of all layers, unlike existing methods that use channel importance only within a single layer. Furthermore, the proposed self-pixel attention submodule uses both the feature maps of the source and target models, unlike existing methods that only utilize the feature map of the source model, so that target feature information can also be considered. The performance of the novel attention submodules has generally improved both in terms of classification accuracy and training convergence speed. However, some experiments using only a single submodule showed reduced classification accuracy. In the future, the proposed method can be extended to an improved transfer learning regularization method based on knowledge distillation through a method of local selection of feature maps of the spatial attention module.

## Figures and Tables

**Table 1:** The purpose and characteristics of target datasets.

Target Dataset	Task	Train Samples	Test Samples	Classes
Stanford Dogs 120	Object Classification	12,000	8580	120
Caltech 256-30	Object Classification	7710	5140	257
Caltech 256-60	Object Classification	15,420	5140	257
CUB-200-2011	Object Classification	5994	5794	200
MIT Indoor 67	Scene Classification	5360	1340	67

**Table 2:** The structure of submodule networks.

Model Name	Layer Type	Parameter	Value
$FC_{SPA}$	Fully connected	Input size	$R^{H\times W}$
		Output size	$R^{H}$
		Activation	ReLU
	Fully connected	Input size	$R^{H}$
		Output size	$R^{H\times W}$
$FC_{GCA}$	Fully connected	Input size	$R^{C_{1}+\cdots +C_{n}}$
		Output size	$R^{(C_{1}+\cdots +C_{n})/r}$
		Activation	ReLU
	Fully connected	Input size	$R^{(C_{1}+\cdots +C_{n})/r}$
		Output size	$R^{C_{1}+\cdots +C_{n}}$

**Table 3:** The selected intermediate layers in the experiments.

Layer Index	ResNet-101	ResNet-50	Inception-V3	MobileNetV2
1	Resnet.layer1.2.conv3	Resnet.layer1.2.conv3	Conv2d_4a_3×3	features.5.conv.2
2	Resnet.layer2.3.conv3	Resnet.layer2.3.conv3	Mixed_5d	features.9.conv.2
3	Resnet.layer3.22.conv3	Resnet.layer3.5.conv3	Mixed_6e	features.13.conv.2
4	Resnet.layer4.2.conv3	Resnet.layer4.2.conv3	Mixed_7c	features.17.conv.2

The criteria of the selected layer is the last convolution layer of the last block in the 4 layers of ResNet model.

**Table 4:** Comparison of top-1 accuracy (%) results with different methods on five datasets.

Model	Dataset	Methods					
		$L^{2}$	$L^{2}-\mathrm{FE}$ [28]	$L^{2}-\mathrm{SP}$ [21]	**DELTA [28]**	**AFA [31]**	**Proposed**
ⓐ	①	82.37%±0.25	87.66%±0.15	87.28%±0.17	87.48%±0.11	88.22%±0.07	88.44%±0.04
	②	78.40%±0.10	62.09%±0.07	79.89%±0.09	80.04%±0.13	80.30%±0.03	80.62%±0.08
	③	84.39%±0.41	83.18%±0.13	85.72%±0.10	85.49%±0.16	86.15%±0.06	86.42%±0.01
	④	86.69%±0.23	83.64%±0.10	87.72%±0.15	86.94%±0.07	87.83%±0.03	87.94%±0.06
ⓑ	⑤	83.06%±0.19	82.00%±0.10	83.70%±0.17	84.06%±0.08	84.34%±0.10	84.82%±0.13

Model ⓐ: ResNet-101, ⓑ: ResNet-50; Dataset ①: Stanford Dogs 120, ②: CUB-200-2011, ③: Caltech 256-30, ④: Caltech 256-60, ⑤: MIT Indoor 67.

**Table 5:** Comparison of top-1 accuracy (%) results with different submodules on five datasets.

Model	Dataset	Modules			
		**AST [31]**	**ACT [31]**	**SPA**	**GCA**
ResNet-101	Stanford Dogs 120	88.12%±0.08	88.06%±0.04	88.29%±0.07	88.18%±0.04
	CUB-200-2011	80.37%±0.16	80.29%±0.12	80.47%±0.14	80.48%±0.15
	Caltech 256-30	85.89%±0.06	85.69%±0.07	85.98%±0.09	86.14%±0.09
	Caltech 256-60	87.20%±0.12	87.02%±0.19	87.30%±0.11	87.87%±0.08
ResNet-50	MIT Indoor 67	84.36%±0.09	84.03%±0.14	84.18%±0.13	84.72%±0.06

**Table 6:** Effects of reduction rate *r* on global channel attention module.

Model	Dataset	Reduction Rate *r*			
		**4**	**8**	**16**	**32**
ResNet-101	Caltech 256-30	86.14%±0.09	86.06%±0.04	86.06%±0.21	86.05%±0.16

**Table 7:** Effects of training order of proposed submodules.

Model	Dataset	Methods	
		**SPA**→**GCA**	**GCA**→**SPA**
ResNet-101	Stanford Dogs 120	88.44%±0.04	88.23%±0.13
	CUB-200-2011	80.62%±0.08	79.93%±0.05
	Caltech 256-30	86.42%±0.01	86.31%±0.08
	Caltech 256-60	87.94%±0.06	88.05%±0.08
ResNet-50	MIT Indoor 67	84.82%±0.13	84.30%±0.07

## Data Availability

The pretrained models using ImageNet and Places 365 source data for transfer learning are available at pytorch pre-trained models (https://pytorch.org/vision/0.8/_modules/torchvision/models/resnet.html, accessed on 1 March 2022) and https://github.com/CSAILVision/places365 (accessed on 1 November 2022), respectively. The dataset on Stanford Dogs 120 is available at http://vision.stanford.edu/aditya86/ImageNetDogs/ (accessed on 1 November 2022). The Caltech-UCSD Birds-200-2011 (CUB-200-2011) and Caltech 256 dataset are available at https://www.vision.caltech.edu/datasets/ (accessed on 1 November 2022). The MIT Indoor 67 dataset is available at https://web.mit.edu/torralba/www/indoor.html (accessed on 1 November 2022).
